# Denileukin Diftitox (Ontak) as Maintenance Therapy for Peripheral T-Cell Lymphomas: Three Cases with Sustained Remission

**DOI:** 10.1155/2015/123756

**Published:** 2015-07-09

**Authors:** Alejandra C. Fuentes, Ellen Szwed, Cathy D. Spears, Sandeep Thaper, Long H. Dang, Nam H. Dang

**Affiliations:** ^1^Department of Internal Medicine, University of Florida, Gainesville, FL 32610, USA; ^2^Division of Hematology/Oncology, Department of Internal Medicine, University of Florida, Gainesville, FL 32610, USA; ^3^Florida Cancer Specialists and Research Institute, Leesburg, FL 34748, USA

## Abstract

Peripheral T-cell lymphomas (PTCL) are rare but markedly aggressive forms of non-Hodgkin's lymphoma (NHL). They carry a poor prognosis, with current therapeutic approach being generally ineffective. The most employed first-line treatment is CHOP (cyclophosphamide, doxorubicin, vincristine, and prednisone), which still results in high rates of relapses. Denileukin diftitox is a fusion protein combining the cytotoxic portion of the diphtheria toxin and the receptor-binding domain of the interleukin-2 (IL-2) molecule, thereby targeting cells expressing the IL-2 receptor, including both T-cell and B-cell lymphomas. It has been approved for the treatment of cutaneous T-cell lymphomas, and it has documented activity in PTCL both as a single agent and as part of combination therapy. This report documents three cases of PTCL where denileukin diftitox has been used as long-term maintenance therapy after complete remission was achieved. While the overall survival rate of patients with advanced stage, refractory PTCL is generally poor (with median overall survival of 5.5 months), the three patients described in this report are all experiencing an ongoing complete remission for more than four years.

## 1. Introduction

Non-Hodgkin's lymphomas (NHL) are a heterogeneous group of lymphoproliferative disorders. Approximately 71,850 new cases of NHL and 19,790 deaths are projected for 2015, accounting for 4% of new cases and 3% of cancer deaths [1]. Although only about 15–20% of NHL cases originate from the T-cell lineage, T-cell non-Hodgkin's lymphomas are generally more aggressive and have a poorer prognosis than their B-cell counterpart, with decreased short-term survival and frequent relapses.

Peripheral T-cell lymphomas (PTCL) comprise most of the cases of T-cell NHL. The classification and treatment options for PTCL have advanced since the REAL Classification recognized PTCL as a specific entity independent from the B-Cell lineage in 1994 [2]. Due to the rarity of PTCL and lack of randomized controlled trials, there is no consensus on first-line therapy. CHOP (cyclophosphamide, doxorubicin, vincristine, and prednisone) has been widely used, largely based on results extrapolated from studies on aggressive B-cell lymphomas. However, CHOP has failed to improve survival and often culminates with frequent relapses, with a 5-year overall survival rates ranging around 32–35% [3, 4]. Studies comparing intensive therapies to CHOP have not demonstrated improved overall survival [5]. These findings highlight the crucial need for improved treatment strategies. In the past few years, advances in molecular biology have identified different cell surface molecular markers, leading to promising novel therapies that are currently under ongoing investigation and are being used as salvage therapy for relapsed disease [3, 4].

Denileukin diftitox (DD) is a recombinant cytotoxic fusion protein that binds the enzymatically active portion of the diphtheria toxin to the receptor-binding domain of the interleukin-2 molecule, thereby targeting cells producing the IL-2 receptor. These cells can derive from multiple lineages, including B and T lymphocytes, macrophages, and natural killer cells. Previous work has demonstrated that DD exhibited significant single-agent activity and was well tolerated in both T-cell and B-cell malignancies, as well as CD25+ and CD25− tumors [6–8].

In 2008 we reported the first case of prolonged remission with long-term maintenance therapy with DD in a patient with advanced stage, relapsed/refractory PTCL [9]. This patient is presently experiencing an ongoing complete remission of more than 9 years (ND, personal communications). To date, no other such cases had been described. This current report describes three additional cases of patients with advanced stage PTCL in which DD was used successfully as long-term maintenance therapy to maintain ongoing remission, hence illustrating the exciting potential of this novel drug as an agent that can be used as maintenance therapy in selected cases of PTCL.

## 2. Case 1

The patient is a 51-year-old African-American female with past medical history significant for Hepatitis B and with relapsed/refractory Stage IVB subcutaneous panniculitis gamma delta T-cell lymphoma. In November 2004 she first came to local medical attention with a firm, nontender 4 × 2 cm growth in her forehead and a smaller one in her left anterior shoulder. Biopsy of the forehead nodule revealed atypical T-cell proliferation with necrosis, and flow cytometry studies demonstrated the presence of a population of CD3+, CD4+, CD8+, CD5+, and CD56− cells.

Her complete blood count at that time revealed WBC 4,200 per mm^3^ (normal differential), Hgb. 10.5 g/dL, Hct. 32.2%, and platelets 297 K/mm^3^. Bone marrow studies showed normal aspirate and core biopsy, with no evidence of abnormal cells. She was diagnosed with an inflammatory reaction of unclear etiology and was placed on a prednisone taper for 6 months, which resulted in clinical improvement and resolution of her nodules by April 2005. Her local dermatologist prescribed Plaquenil after the prednisone taper, based on a presumed diagnosis of lymphoid dermatitis.

In November 2006, she experienced a recurrence of subcutaneous nodules in her right eye, left forearm, and left chest area, each measuring approximately 1 cm in size. Her WBC was 4,900 per mm^3^ with predominant lymphocytosis at 41%, Hgb. 12.2 g/dL, Hct. 36.9%, and platelets 349 K/mm^3^. Computed tomography (CT) scan also revealed a new subcutaneous soft tissue density measuring 3.8 × 2.3 cm in the left anterior abdomen. Biopsy of the abdominal mass showed morphologically atypical lymphocytes that appeared to rim adipocytes in a manner described for panniculitis-like T-cell lymphoproliferative disorders, with Ki-67 proliferation marker expressed in many of the cells. Immunohistochemical stains showed a predominance of CD3+/CD5+ T-cells, comprised of a mixture of CD4+ and CD8+ cells, with a few background CD20+ B-cells. CD56, CD57, CD30, ALK-1, and CD10 markers were negative. CD25 was not tested. However, a clonal population was not identified by flow cytometry and T-cell gene rearrangement analysis was negative. By January 2007, her nodules were increasing in size and in light of her clinical picture she was treated for presumed lymphoma with 6 cycles of CVP (cytoxan, vincristine, and prednisone) every 3 weeks. She tolerated the treatment well and her nodules resolved. Follow-up PET scan in July 2007 showed abnormal FDG activity in the head of the pancreas, and all labs were otherwise normal. Patient was thereafter lost to follow-up.

In May 2010, she presented to the emergency department with bilateral lower extremity swelling, upper extremity nodules, fevers, night sweats, abdominal pain, vomiting, and diarrhea for one month. CT imaging revealed recurrent multiple indurated lesions in the left flank. Biopsy of a right arm nodule showed lobular panniculitis and focal fat necrosis with involvement by an atypical T-cell infiltrate. Molecular studies were positive for a monoclonal rearranged band of T-cell receptor gamma gene and flow cytometry revealed increased level of gamma/delta T-cells at approximately 40%, consistent with subcutaneous panniculitis-like T-cell lymphoma. Bone marrow biopsy showed normal cellularity (80–90%) with multilinear hematopoiesis and no evidence of lymphoma. She received 3 cycles of ICE (ifosfamide, carboplatin, and etoposide) combination therapy, but her disease continued to progress with increasing number of subcutaneous nodules and continuing constitutional symptoms. She then received two cycles of ESHAP (etoposide, methylprednisolone, cytarabine, and cisplatin) with mixed response based on CT scan with no evidence of adenopathy but increase in nodularity and size of the anterior abdomen subcutaneous nodule. She was evaluated for stem cell transplantation, but she needed to attain at least partial remission before being a candidate.

**Figure 1:**
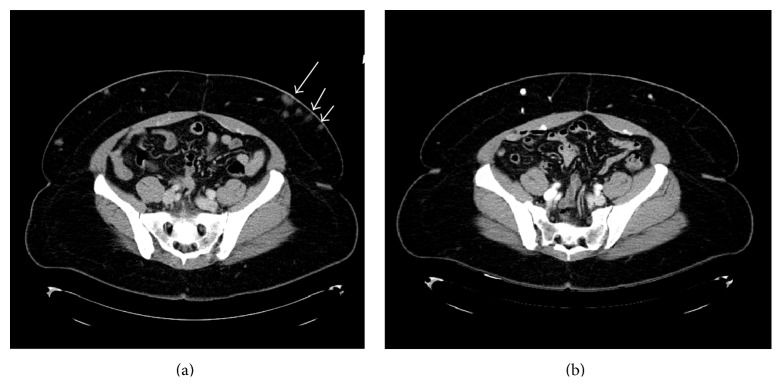
Case 1: CTs of the abdomen illustrating (a) subcutaneous nodules prior to initiation of denileukin diftitox and (b) resolution of the subcutaneous nodules after treatment with denileukin diftitox.

Since she had multiple relapses despite treatment with CVP, ICE, and ESHAP combination therapies, she was started on treatment with DD in December 2010. The treatment was administered intravenously at 18 *μ*g/kg daily for 5 days every 4 weeks, with steroid premedication. After a total of 8 cycles, CT imaging confirmed resolution of her subcutaneous nodules (Figure 1). Due to the clinical and radiological response, she then received an additional 8 cycles of DD as maintenance therapy, given at 18 *μ*g/kg daily for 5 days every 8 weeks, which she continued to tolerate well without side effects. Her performance status remained zero throughout the entirety of her disease course and treatment, and the patient has now achieved ongoing complete remission of more than 4 years, as documented by periodic surveillance scans, physical exams, and routine blood tests.

## 3. Case 2

The patient is a 71-year-old Caucasian male with past medical history of type-2 diabetes mellitus, deep vein thrombosis with inferior vena cava filter placement in 2010, and newly diagnosed stage IVB peripheral T-cell lymphoma.

He first presented to an outside institution in April 2010 with complaints of progressive groin adenopathy, leg swelling, and fatigue for several months. His CBC at that time revealed WBC 6,300 per mm^3^ (with 77.0% neutrophils and 9.6% lymphocytes), Hgb. 13.8 g/dL, Hct. 44.3%, and platelets 252 K/mm^3^. CT imaging demonstrated disease on both sides of the diaphragm with a 1.5 cm left axillary node and subcarinal node, as well as diffuse lymphadenopathy in the retroperitoneum, pelvis, and left groin. A lymph node biopsy was positive for peripheral T-cell lymphoma, NOS. Bone marrow biopsy did not show definitive morphologic involvement with a T-cell lymphoproliferative disorder, but T-cell clonality analysis was positive for a clonal T-cell receptor beta gene rearrangement. Flow cytometric analysis of the bone marrow detected a small population of T-cells (5%), which had an inverted CD4 : CD8 ratio at 0.3 : 1; otherwise no aberrant antigen expression was detected. CD25 was not tested.

He was started on treatment with CHOP combination chemotherapy and achieved complete remission after 6 cycles by August 2010, as documented by end-of-treatment CT scans showing complete resolution of adenopathy and repeat bone marrow biopsy indicating complete clearance of tumor cells. CBC at this time revealed WBC 3,000 per mm^3^ (with 70.3% neutrophils and 15.5% lymphocytes), Hgb. 14.9 g/dL, Hct. 43.6%, and platelets 138 K/mm^3^.

He was not a candidate for consolidation with stem cell transplantation due to his age. In view of the high risk of relapse and poor prognosis associated with stage IVB PTCL, the patient opted for maintenance therapy with DD, starting with the first cycle in December 2010. DD was administered intravenously at 18 *μ*g/kg daily for 5 days every 4 weeks, for the first eight cycles, and then additional 8 cycles were given at 18 *μ*g/kg daily for 5 days every 8 weeks. He completed a total of 16 cycles without any side effects. He experienced decreased fatigue and improved performance status from 1 to 0. The patient has now maintained an ongoing complete remission of greater than 4 years as documented by periodic surveillance scans, bone marrow biopsies, physical exams, and routine blood tests.

## 4. Case 3

The patient is a 27-year-old Hispanic female with past medical history of coarctation of the aorta, with Stage IIIA CD30+ anaplastic large cell lymphoma, null type, positive for ALK-1 (anaplastic lymphoma kinase-1).

She first presented in August 2010 to an outside institution with fevers, night sweats, paresthesias, 15 lb weight loss, and bilateral supraclavicular adenopathy with mild swelling in her groin. CT scans revealed extensive mediastinal, pelvic, and retroperitoneal lymphadenopathy with accompanying ascites and mild splenomegaly. The largest nodes were a mesenteric lymph node measured 2.3 cm and a right inguinal lymph node 3 cm. Right inguinal lymph node biopsy revealed anaplastic large cell lymphoma of null type, ALK-1 positive. Immunohistochemical stain was strongly positive for ALK-1, CD4, CD30, CD25, and epithelial membrane antigen (EMA) at 60%, mildly positive for CD45, and negative for CD3, CD20, and CD8. The pattern of ALK-1 immunostain was diffuse cytoplasmic with peripheral intensification, suggesting ALK partner with TPM3 (t(1; 2)(q25; p23)). Bone marrow studies showed a normal aspirate and core biopsy, without evidence of abnormal cells. Her WBC was 9,700/mm^3^, Hgb. 11.2 g/dL, Hct. 33.4%, and platelets 285 K/mm^3^.

She received CHOP combination chemotherapy with initial response and resolution of palpable lymphadenopathy after three cycles. However, two weeks after her third cycle in October 2010, she noted increasing cervical adenopathy. A repeat CT scan revealed left cervical, left supraclavicular and right inguinal lymphadenopathy, with the largest inguinal node measuring 1.7 cm with heterogenous appearance. Cervical lymph node biopsy revealed persistent anaplastic large cell lymphoma with the same immunophenotype. Her CBC showed WBC 3,400/mm^3^, Hgb. 11.5 g/dL, Hct. 35.9%, and platelets 218 K/mm^3^. She was referred to our institution in November 2010, where she received four cycles of ICE (ifosfamide, carboplatin, and etoposide) salvage chemotherapy, finishing in December 2010. Follow-up CBC in January 2011 showed WBC 3,900/mm^3^, Hgb. 9.1 g/dL, Hct. 25.5%, and platelets 72 K/mm^3^, and CT scan at that time demonstrated remission with no evidence of nodal disease.

She declined consolidation with stem cell transplantation and opted for maintenance therapy with DD, which was initiated in February 2011. She received 6 cycles administered intravenously at 18 *μ*g/kg daily for 5 days every 4 weeks, and then additional 4 cycles were administered intravenously at 18 *μ*g/kg daily for 5 days every 8 weeks. She received a total of 10 cycles by March 2012 and was then lost to follow-up. CT imaging after cycles 3, 6, and 10 were negative for recurrence of disease. Associated side effects included fatigue and weight gain after the first cycle and bilateral leg swelling after the fifth cycle (improved with furosemide administration), as well as anasarca and lower extremity deep vein thrombosis (DVT) after the tenth cycle.

She returned in January 2013 to reestablish care and has continued to show no recurrence of disease. The patient has now maintained an ongoing complete remission of greater than 4 years as documented by periodic surveillance scans, physical exams, and routine blood tests.

## 5. Discussion

Although PTCL only account for 6% of NHLs and are relatively rare when compared to their B-cell counterparts, they tend to be aggressive and are associated with higher international prognostic index (IPI) scores. Relapses after treatment with CHOP, the most employed therapy, are common, leading to poor prognosis and short-term survival, with 5-year overall survival (OS) rates ranging around 32–35% [3, 4]. Patients with relapsed or refractory PTCL have a reported median progression-free survival of 3.1 months and a median OS of 5.5 months [10]. In contrast, after using DD as maintenance therapy, the cases presented in this report have achieved ongoing sustained remission for over 4 years. DD as maintenance therapy has only been reported in one prior case [9], and this patient is presently experiencing sustained remission for 9+ years (ND, personal communications). To our knowledge, these are the only four patients who have received DD as maintenance therapy, and all four of them have achieved remarkable results. As exemplified in these cases, this novel targeted therapy may provide an alternative to stem cell transplantation (SCT), whose role as consolidation therapy in PTCL remains controversial, with some studies supporting its efficacy and others suggesting lack of improved outcomes [11–14]. Additionally, as evidenced in this report, not all patients meet criteria for SCT.

The efficacy of DD as salvage therapy in otherwise refractory PTCL has already been well documented in a phase II trial [6]. It has also been demonstrated that combination therapy with DD and CHOP is effective, with OS rate of 63.3% and median duration of response of 30 months, as opposed to historical data of 32–35% and 12 months, respectively, with CHOP alone [15]. However, it is important to note that previous publications on DD have studied its activity with limited cycles of treatment (a maximum of 8 cycles), with disease eventually recurring. In contrast, the cases presented here demonstrate the efficacy of DD when it is used as maintenance therapy (over a span of 1-2 years), which can result in long-term remission, even long after DD has been discontinued.

In our first case, although stage IVB subcutaneous panniculitis gamma delta T-cell lymphoma typically results in poor survival, DD was successful as salvage therapy after the disease had relapsed with CVP, ICE, and ESHAP and subsequently served as maintenance therapy, resulting in sustained remission. Maintenance therapy in our second case also resulted in sustained remission, despite the fact that the patient had stage IVB PTCL, NOS, which is typically associated with poor prognosis. Although ALK-1 positive anaplastic large cell lymphoma is generally associated with improved prognosis due to superior response rates to CHOP-like regimens [5, 16], our patient in the third case had stage III disease that was refractory to CHOP, rendering a poor prognosis. However, she also has experienced continuing sustained remission for more than four years following long-term therapy with DD. Our first two cases tolerated the drug exceedingly well and case 3 exhibited some adverse events (anasarca and DVT). While DD can be associated with adverse reactions including capillary leak syndrome, infusion reactions, and visual changes, among others, it is not associated with cumulative toxicity or clinically significant myelosuppression [6].

Although a specific mechanism remains unclear, it is our hypothesis that the activity of DD as long-term maintenance therapy involves its ability to potentially regulate the immune system. Specifically, T-regulatory cell population is CD25+ and long-term DD therapy may depress the level of CD25+ T regulatory cell populations, resulting in increased immune activity against the cancer cells and long-term remission. Such immune-surveillance activity by anti-CD25 agents has been described in prior studies [17–20]. This may explain how DD is effective against a heterogeneous group of lineages, including T-cell and B-cell malignancies, and both CD25+ and CD25− tumors [6–8].

Given the demonstrated efficacy of DD as maintenance therapy and its general tolerability in advanced stage PTCL with generally poor prognosis, our findings can have significant implications. Additional studies would need to be conducted in a formal clinical trial setting to firmly establish the role of DD as potential maintenance therapy in PTCL.
